# N^6^-methyladenine: a potential epigenetic mark in eukaryotes

**DOI:** 10.18632/oncotarget.4684

**Published:** 2015-06-26

**Authors:** Shoujun Huang, Dahua Chen

**Affiliations:** State Key Laboratory of Reproductive Biology, Institute of Zoology, Chinese Academy of Sciences, Beijing, China

**Keywords:** Chromosome Section, DNA methylation, N^6^-methyladenine, epigenetic mark, eukaryotes

Methylation modifications in DNA in the forms of 5-methylcytosine (5mC) and N^6^-methyladenine (6mA) are one of the most important epigenetic marks that have been proposed to regulate gene expression and control numerous cellular and biological processes. The prevailing view is that, while 5mC serves as the predominant type, if not the only type, of methylated base in mammals to regulate gene expression and maintain chromatin architecture [1], 6mA is important in bacteria to control a number of biological functions, such as DNA replication and repair, gene expression, and host-pathogen interactions. In particular, 6mA is essential for the viability of some bacterial strains [2].

Previous studies have reported that 6mA is not only present at considerable levels in genomic DNA from a number of unicellular eukaryotes, but also is detected in some plant DNA. However, 6mA has been shown to be present at extremely low levels in most higher eukaryotes and particularly in mammals. Thus, whether 6mA occurs widely and what its potential roles are in higher eukaryotic cells remain unknown [2]. We hypothesize that, if 6mA does play a role, the potential introduction of this modification by methyltransferases could be reversed by a demethylase-mediated demethylation process, given the low levels of 6mA detected in higher eukaryote DNA. Thus, the detection of 6mA demethylases in the genomes of higher eukaryotes is critical to test the model we have hypothesized.

We and others recently provided compelling evidence showing that 6mA modification occurred in DNA from three different eukaryotes, *Chlamydomonas reinhardtii*, *Caenorhabditis elegans*, and *Drosophila melanogaster*, and that it played conserved epigenetic roles in regulating gene expression [3-5]. In *Drosophila*, we found that 6mA was highly present at the very early embryonic stage, but very low levels were present at the late embryonic stage, suggesting that 6mA modification may be dynamic during embryogenesis. Indeed, further evidence revealed that the dynamics of 6mA modification were tightly regulated by a DNA 6mA demethylase (DMAD), the *Drosophila* Tet-like protein. Genetic analyses revealed that loss of *DMAD* led to a significant increase in levels of 6mA in multiple adult tissues, and the adult mutant fly displayed strong developmental defects, supporting the notion that DMAD suppressed DNA 6mA modification *in vivo*, and DMAD-mediated 6mA demethylation was essential for development and tissue homeostasis. Moreover, 6mA DNA immunoprecipitation sequencing (MeDIP-seq) analyses revealed that DMAD determined 6mA distribution in transposon regions in *Drosophila* ovary DNA and that the regulation of DMAD demethylase activity was correlated with transposon expression. Together, our findings suggested that 6mA modification acts as an epigenetic mark that likely regulates gene expression [3].

In line with our findings, Shi and colleagues showed that 6mA modification in *C. elegans* was regulated by a DNA demethylase, NMAD-1, and a potential DNA methyltransferase, DAMT-1. Notably, the reciprocal regulation between 6mA and histone modification H3K4me2 was shown to contribute to trans-generationally epigenetic control in the worm. By sequencing analyses, Shi and colleagues identified two motifs, GAGG and AGAA, as 6mA modification hotspots. Both these motifs indicated that 6mA occurred only on one strand of DNA in a local context in *C. elegans* [4]. Interestingly, another study by He and colleagues showed that 6mA had a bimodal distribution pattern around the transcription start sites (TSSs) of actively transcribed genes in *Chlamydomonas* (green algae). A genome level nucleosome footprinting analysis indicated that 6mA was preferentially located at the linker DNA of chromatin and may regulate nucleosomes positioning near the TSSs in *Chlamydomonas*. These findings together imply that 6mA can function as an epigenetic mark in eukaryotes [5].

However, the mechanisms that link 6mA to epigenetic control in higher eukaryotes are only just beginning to be identified. A number of important issues regarding the role of the 6mA modifications are yet to be addressed. 1) Although the presence and function of 6mA in *Chlamydomonas*, *C. elegans*, and *Drosophila* have been determined, it is still unknown whether 6mA modification occurs in vertebrates, especially in mammals. 2) The biochemical properties of 6mA methyltransferases need to be explored further. Whether METTL4 has a conserved role in regulating enzymatic installation of 6mA needs to be determined. In addition, the identification of other 6mA methyltransferases is important for understanding how 6mA is regulated. 3) Enzymes responsible for 6mA demethylation in different organisms need to be identified. DMAD was shown to control 6mA demethylation in Drosophila. In future studies, it will be interesting to search for double-stranded beta-helix (DSBH)-domain-containing dioxygenases responsible for 6mA demethylation in mammals. 4) The genomic distribution of 6mA in different organisms needs to be investigated. 5) In a previous study, members of the YTH domain-containing family were found to “read” RNA m6A signals and regulate RNA degradation and translation. A similar method could be used to identify a DNA 6mA “reader” in higher eukaryotes. 6) The relationship between 6mA and human diseases such as cancer, require further clarification. This relationship is discussed below.

DNA damage-inducing reagents are very important cancer treatment drugs. In some cancers, such as bladder, prostate and pancreatic cancers, members of the ALKBH family have been shown to have increased expression levels, which can lead to DNA damage resistance in tumors, and contribute to cancer cell proliferation and chemotherapy resistance [6, 7]. Interestingly, we found that human primary tumor samples had extremely low levels of 6mA staining (about 10% of adjacent tissue 6mA level; our unpublished data) compared with adjacent noncancerous tissue samples. We reasoned that some of the mammalian ALKBH family members may possess either specific or non-specific demethylation activity against 6mA. Thus, at least in some ALKBH-overexpressing tumors, loss of 6mA modification could be a novel hallmark of cancer. In future studies, the 6mA levels in different types of cancers need to be further analyzed.
